# ANKRD22 enhances breast cancer cell malignancy by activating the Wnt/β-catenin pathway via modulating NuSAP1 expression

**DOI:** 10.17305/bjbms.2020.4701

**Published:** 2021-06

**Authors:** Yange Wu, Hongxia Liu, Yufeng Gong, Bo Zhang, Wenxiu Chen

**Affiliations:** Department of Pathology, Pingshan District People’s Hospital of Shenzhen, Pingshan General Hospital of Southern Medical University, Shenzhen, China

**Keywords:** Breast cancer, MDA-MB-415, ANKRD22, NuSAP1, Wnt/β-catenin pathway

## Abstract

Breast cancer is one of the most prevalent malignancies in women worldwide. Although great advancements have been achieved in the diagnosis and treatment of breast cancer, the prognosis of patients with breast cancer is still poor due to distal recurrence and metastasis after surgery. This study aimed to assess the role of ankyrin repeat domain 22 (ANKRD22) in the progression of breast cancer and investigate the molecular mechanism. Using immunohistochemistry, we demonstrated that the expression level of ANKRD22 in human breast cancer tissues was significantly higher than that in normal breast tissues. *ANKRD22* knockdown inhibited the proliferation, invasion, and epithelial-mesenchymal transition (EMT) of breast cancer cells, as confirmed by BrdU, colony formation, transwell, and immunoblot assays. Immunoblot assays further indicated that ANKRD22 regulated the expression of nucleolar and spindle-associated protein 1 (NuSAP1) and then caused the activation of Wnt/β-catenin signaling pathway. Moreover, overexpression of *NUSAP1* reversed the inhibitory effects of *ANKRD22* knockdown on the proliferation, invasion, and EMT of breast cancer cells. In summary, this study demonstrated that ANKRD22 enhanced breast cancer cell malignancy by activating the Wnt/β-catenin pathway via modulating NuSAP1 expression, which might shed light on new therapeutic approaches for breast cancer.

## INTRODUCTION

Breast cancer is one of the most prevalent malignancies in women worldwide [1]. More than 1.2 million cases are diagnosed with breast cancer worldwide and about 500,000 women die from this disease per year [2]. Despite great progress has been achieved in the diagnosis and treatment of breast cancer, the overall survival is still poor due to the distal recurrence and metastasis after surgery [3]. Immunotherapy and targeted therapy have emerged as potential treatment methods for breast cancer [4]. Multiple targets have been identified such as vascular endothelial growth factor, epithelial membrane protein 2, long noncoding RNAs, and so on [5,6]. However, more therapeutic targets are also urgently needed.

Ankyrin repeat domain 22 (ANKRD22), a member of the ankyrin repeat (ANKR) family, is constitutively expressed in normal digestive tract epithelium and in tumor cells [7]. ANKR family proteins have been reported to play vital roles in cancer development and progression [8-10]. For example, ANKRD1 promotes renal cancer cell growth, while ANKRD12 has the opposite effect on colorectal cancer [11,12]. Recently, studies demonstrated that ANKR protein facilitated the development of non-small cell lung cancer (NSCLC), but suppressed the development of prostate cancer, suggesting that ANKRD22 exerts pro-tumor or anti-tumor effects in different tumor types [9]. In particular, ANKRD22 has been reported to be a marker of pancreatic cancer. However, its function in breast cancer is still unclear.

Nucleolar spindle-associated protein 1 (NuSAP1) is a microtubule-binding protein that participates in the regulation of mitotic progression and spindle formation and stability [13]. Recent studies have found that NuSAP1 was overexpressed in several types of cancers such as prostate cancer, colorectal cancer, and astrocytoma [14-16]. NuSAP1 also served as a key regulator of breast cancer progression, and it contributed to cell proliferation and invasion [17]. High expression of NuSAP1 was correlated with poor prognosis of breast cancer and melanoma [18]. In view of the potentially important role of NuSAP1 in the development of breast cancer, along with the predicted relationship between NuSAP1 and ANKRD22 from the Cancer Genome Atlas (TCGA) database, it may be possible that ANKRD22 affects the development of breast cancer via NuSAP1. However, the specific regulatory mechanism is unknown.

In this study, we demonstrated that the expression level of ANKRD22 in breast cancer tissue was significantly higher than that in normal breast tissue. ANKRD22 knockdown suppressed the proliferation, invasion, and epithelial-mesenchymal transition (EMT) of breast cancer cells. Further research indicated that ANKRD22 regulated the expression of NuSAP1 and activated the Wnt/β-catenin signaling pathway. The inhibitory effects of *ANKRD22* knockdown on the proliferation, invasion, and EMT of breast cancer cells were reversed by NuSAP1 overexpression. Therefore, ANKRD22 might be a promising novel therapeutic target for the treatment of breast cancer.

## MATERIALS AND METHODS

### Ethics statement

All studies were conducted in accordance with the standards from the Ethics Committee of Pingshan District People’s Hospital of Shenzhen, and also in accordance with the Declaration of Helsinki published in 1964 and all subsequent revisions. Informed consent was obtained from each patient.

### Tissue specimens

Breast cancer tissues were surgical specimens with complete clinical-pathological data collected from the l Pingshan District People’s Hospital of Shenzhen (53 cases). Fresh normal breast tissues were obtained from breast reduction surgery or quadrantectomy (17 cases).

### Cell culture and transfections

Human breast cancer MDA-MB-415 (ATCC^®^ HTB-128) [19], MDA-MB-468 (ATCC^®^ HTB-132), MCF7 (ATCC^®^ HTB-22), and MDA-MB-361 (ATCC^®^ HTB-27) [19] cell lines were purchased from ATCC (Maryland, USA) and grown in RPMI-1640 medium (Hyclone,Logan, Utah, USA) with 10% fetal bovine serum [FBS] (Gibco, New York, USA) at 37°C in a humidified atmosphere containing 5% CO_2_. Cells were transfected with short hairpin RNA (shRNA) plasmids targeting *ANKRD22* by Lipofectamine 2000 (11668019, Invitrogen, USA).

### Immunohistochemistry (IHC) assay

Breast cancer and normal tissues were fixed using 4% paraformaldehyde (PFA) for 30 minutes at room temperature, then blocked with 2% bovine serum albumin (BSA, Sigma, USA) for 20 minutes. Subsequently, sections were incubated with ANKRD22 antibody (1:100, Novus Biologicals, USA) for 2 hours at room temperature. All sections were incubated with horseradish peroxidase (HRP, Abcam, Cambridge, UK) secondary antibody (Abcam, UK) for 1 hour and 30 minutes, added with diaminobenzidine, and then counterstained with hematoxylin.

The expression level of ANKRD22 was divided into four groups based on the staining intensity (0 for negative staining; 1 for low staining; 2 for medium level; 3 for high level). Meanwhile, the proportion of stained cells was as follows (0 for 0% staining cells; 1 for 1–30% staining cells; 2 for 31–60% staining cells; 3 for 61–100% staining cells). The score of staining intensity × score of stained cells percentage =0 or =1 was considered as negative staining, 2–4 was considered as low staining, and over 4 was considered as ANKRD22 high expression. ANKRD22 was located in the cytoplasm and colored in brown-yellow, and its expression was classified by IHC staining score.

### Quantitative reverse transcription polymerase chain reaction (qRT-PCR)

Total RNA was extracted from cells or tissues with Trizol reagent (Invitrogen, Waltham, MA, USA) and used for the first-strand cDNA synthesis with the Reverse Transcription System (Roche, Indianapolis, IN, USA) following the manufacturer’ s protocol. Glyceraldehyde 3-phosphate dehydrogenase (GAPDH) was used as a control. The sequences of primers used were as follows: *GAPDH*, forward 5’-CGACCACTTTGTCAAGCTCA-3’ and reverse 5’-GGTTGAGCACAGGGTACTTTATT-3’; *ANKRD22*, forward 5’-GACCCCACAATAAAGAATAAGC-3’ and reverse 5’-CCCACAGACCAAAAGTCTAAAA-3’; *VIM*, forward 5’-GACGCCATCAACACCGAGTT-3’ and reverse 5’-CTTTGTCGTTGGTTAGCGGT-3’; *TWIST1*, forward 5’-GTCCGCAGTCTTACGAGGAG-3’ and reverse 5’-GCTTGAGGGTCTGAATCTGCT-3’; *CDH1*, forward 5’-ATTTTTCCCTCGACACCCGAT-3’ and reverse 5’-TCCCAGGCGTAGACCAAGA-3’; *CDH2*, forward 5’-AGCCAACCTTAACTGAGGAGT-3’ and reverse 5’-GGCAAGTTGATTGGAGGGATG-3’; and *KRT18*, forward 5’-TTGACAATGCCCGTCTTGCT-3’ and reverse 5’-GCCTTTTACTTCCTCTTCGTGGT-3’. The reaction conditions were as follows: pre-denaturation, 95°C, 3 minutes; denaturation, 95°C, 30 seconds; annealing, 58°C, 30 seconds; extension, 72°C, 30 seconds. There were a total of 35 cycles. The 2-^DDCq^ method was used to quantify the results [20]. The mRNA *ANKRD22* levels were normalized to *GAPDH*.

### Immunoblot assay

Cells were collected and lysed by RIPA buffer and 50 μg proteins were separated by sodium dodecyl sulfate polyacrylamide gel electrophoresis, the proteins were then transferred onto polyvinylidene difluoride membranes and blocked with 5% fat-free milk. The membranes were incubated with primary antibodies for ANKRD22, E-cadherin, N-cadherin, β-catenin, GAPDH, and P84 for 2 hours at room temperature, and then incubated with HRP conjugated secondary antibody (Cell Signaling, 1:1000 dilution, Boston, USA) for 1 hour. The signals were visualized by chemiluminescence (ECL) and detected by Western Blotting Detection system (Tanon, China). The expression of E-cadherin (CDH1), keratin 18 (KRT18), N-cadherin (CDH2), vimentin (VIM), and twist family bHLH transcription factor 1 (TWIST1) was detected through immunoblot assay.

### BrdU assay

Cells were fixed in 4% PFA and permeabilized in phosphate buffered saline with Tween 20 (PBST). BrdU labeling solution (10 µM) was added to cells for 1 hour at 37ºC in a CO_2_ incubator. Then BrdU labeling solution was removed from the cells and washed twice in PBS and for three more times with PBS.

### Transwell assay

*ANKRD22* shRNA plasmids or control were transfected into breast cancer cells for 48 hours and cells were re-suspended in serum-free medium. First, 20% Matrigel was used to coat the upper chambers of filters (8.0 µm membrane pores). Then, about 10^5^ cells in 200 µL of medium were cultured in the upper chambers of the inserts to induce migration toward the bottom chambers containing complete medium with 10% FBS. Twenty-four hours later, cells in the top chamber were taken out by cotton swabs, and the remaining cells were fixed in 4% PFA and stained using 0.2% crystal violet for 30 minutes. Cell number was manually counted.

### Colony formation assay

*ANKRD22*-plasmids or *ANKRD22*-shRNA were transfected into breast cancer cells for 48 hours, and 1000 cells were cultured in a 6-well plate. After 4 weeks, colonies were fixed using PFA for 30 minutes at room temperature and stained with 0.2% crystal violet for 30 minutes, photographed, and colony number was manually counted.

### Statistical analysis

Data were analyzed by GraphPad Prism version 8.0.0 for Windows (GraphPad Software, San Diego, California, USA, www.graphpad.com). All data were represented as mean ± standard deviation (SD) and comparisons between two groups were analyzed by Student’s t-test. The correlation between clinical-pathological characteristics and protein expression was evaluated using χ^2^ analysis. The correlation analysis between *NUSAP1* and *ANKRD22* mRNA levels was performed by GraphPad Prism version 8.0.0. * represents *p* < 0.05, which was considered statistically significant.

## RESULTS

### ANKRD22 expression is upregulated in human breast cancer tissues

**FIGURE 1:**
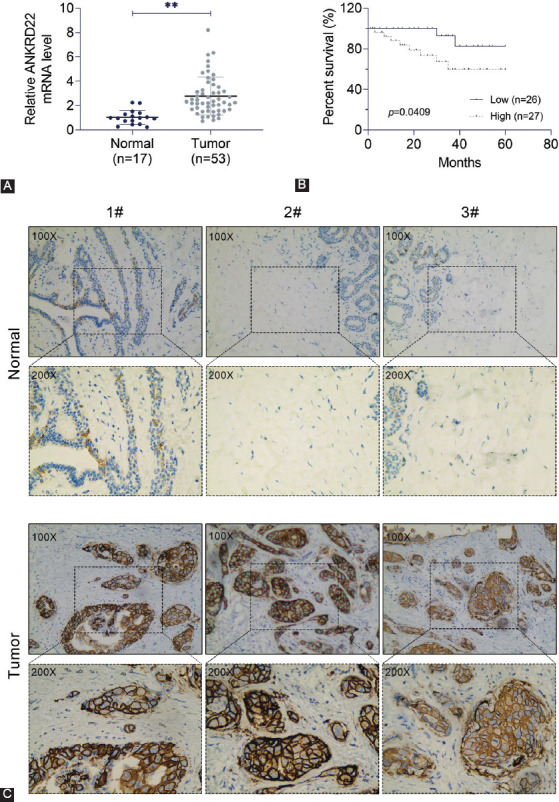
*ANKRD22* expression is upregulated in breast cancer tissues. (A) The mRNA expression level of ANKRD22 in breast cancer tissues and the corresponding normal tissues was detected by quantitative reverse transcription polymerase chain reaction. (B) The correlation between the overall survival and ANKRD22 expression level in breast cancer patients; *p* = 0.0409. (C) Immunohistochemistry assay was performed to detect ANKRD22 expression in human breast cancer and normal tissues, and the representative photographs are shown (×100 and ×200 magnifications, respectively). ANKRD22: Ankyrin repeat domain 22.

To explore the potential effects of ANKRD22 on the progression and development of breast cancer, we analyzed ANKRD22 mRNA expression levels in 70 clinical samples, including 53 breast cancer tissue samples and 17 normal breast tissue samples. The results showed that breast cancer tissues displayed significantly higher expression levels of ANKRD22 than normal breast tissue (*p* < 0.01) (Figure 1A). Patients were classified into ANKRD22 high and low expression groups. Patients with low ANKRD22 expression had a higher survival rate than those with high ANKRD22 expression (*p* = 0.0409) (Figure 1B). Results from IHC assay showed that the expression of ANKRD22 in human breast cancer tissues was higher than that in normal tissues (Figure 1C).

Subsequently, we analyzed the correlations between ANKRD22 expression and clinical-pathological characteristics of patients. The clinical features, including patient age, tumor size, skin involvement, lymph node (LN) metastasis, histologic grade, vessel invasion, tumor grade, and histologic grade, were analyzed. The results (Table 1) showed no significant correlations between ANKRD22 expression and age (*p* = 0.659), tumor size (*p* = 0.318), the tumor status (progesterone [PR], estrogen [ER], and human epidermal growth factor receptor 2 [HER2]) or histological types (Table 1). However, ANKRD22 expression was correlated with LN metastasis (*p* = 0.016*), vessel invasion (*p* = 0.028*), tumor-node-metastasis (TNM) stage (*p* = 0.020*), and tumor grade (*p* = 0.019*).

**TABLE 1:**
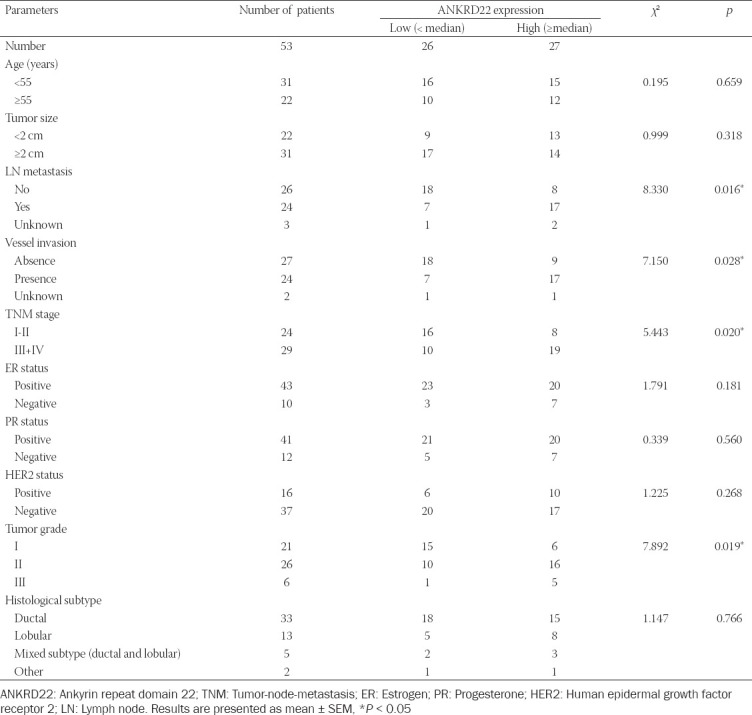
Relationship between ANKRD22 and clinical-pathological parameters

Therefore, our results indicated that the expression level of ANKRD22 was upregulated in breast cancer tissue and associated with the prognosis and clinical-pathological features.

### ANKRD22 knockdown inhibits the proliferation, invasion, and EMT of breast cancer cells

**FIGURE 2:**
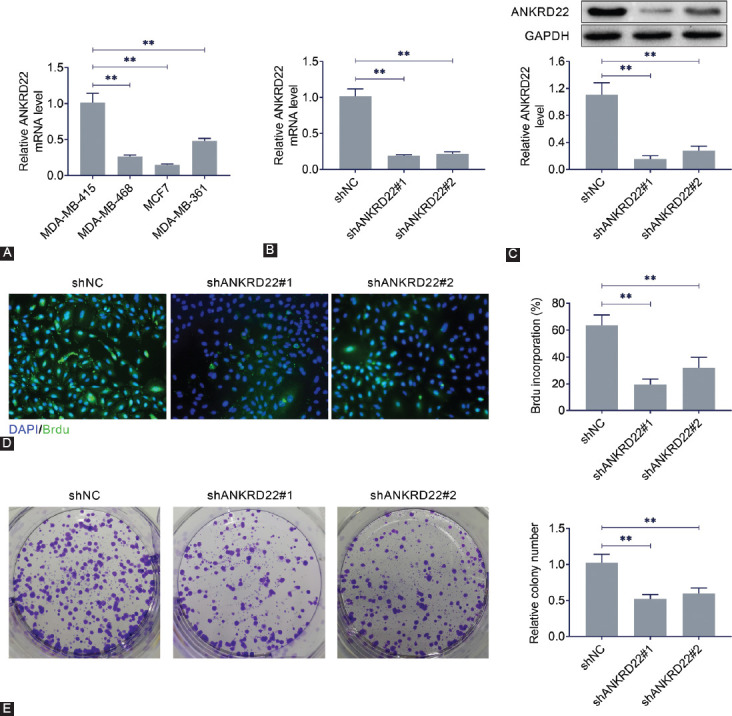
Effects of *ANKRD22* knockdown on the growth and proliferation of breast cancer cell lines. (A) *ANKRD22* expression levels in four different breast cancer cell lines. (B) *ANKRD22* mRNA expression levels in breast cancer cell line MDA-MB-415 after transfection of *ANKRD22* shRNA. (C) *ANKRD22* protein expression levels in breast cancer cell line MDA-MB-415 after transfection with *ANKRD22* shRNA. (D) The effects of *ANKRD22* knockdown on MDA-MB-415 cell proliferation by BrdU assay. (E) Colony formation assay was performed to detect the effect of *ANKRD22* knockdown on MDA-MB-415 cell colony formation. *ANKRD22*: Ankyrin repeat domain 22; shRNA: Short hairpin RNA. Results are presented as mean ± SEM, **p < 0.01.

To explore the potential mechanism of ANKRD22 in breast cancer, ANKRD22 expression was determined in four different breast cancer cell lines. The expression level of ANKRD22 in MDA-MB-415 cells was higher than that in MDA-MB-468, MCF7, and MDA-MB-361 cells (Figure 2A). Then, *ANKRD22* was knocked down by targeted shRNA in MDA-MB-415 cells. qRT-PCR (Figure 2B) and immunoblot (Figure 2C) assays were used to validate the transfection efficiency of *ANKRD22* shRNA in MDA-MB-415 cells.

Afterward, the effects of *ANKRD22* on MDA-MB-415 cell proliferation were validated by BrdU assay. Notably, the proliferative capacity of MDA-MB-415 cells was significantly blocked by *ANKRD22* knockdown, as evidenced by decreased numbers of cells with green fluorescence (Figure 2D). Similarly, colony formation assay showed that *ANKRD22* knockdown inhibited the proliferation of MDA-MB-415 cells (Figure 2E).

**FIGURE 3:**
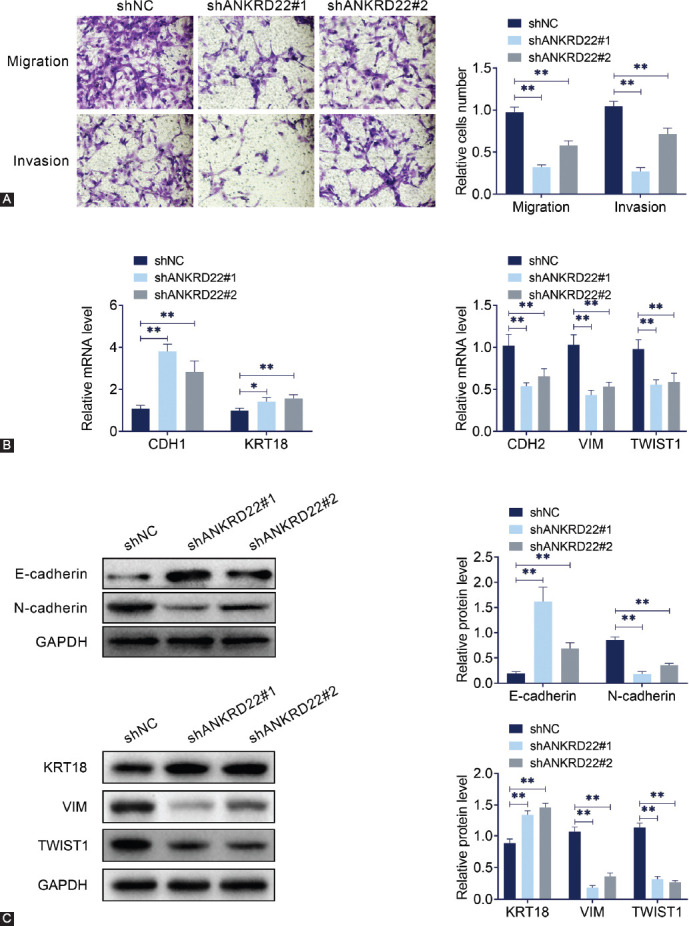
Effects of *ANKRD22* knockdown on invasion and EMT of breast cancer cell lines. (A) The effects of *ANKRD22* knockdown on MDA-MB-415 cell invasion and migration were detected by transwell assay. (B) The mRNA levels of the indicated genes after *ANKRD22* was knocked down. (C) The protein levels of two EMT-related proteins, E-cadherin and N-cadherin, in MDA-MB-415 cells after *ANKRD22* was knocked down. *ANKRD22*: Ankyrin repeat domain 22; EMT: Epithelial-mesenchymal transition; KRT18: Keratin 18; CDH1: E-cadherin; CDH2: N-cadherin; VIM: Vimentin; TWIST1: Twist family bHLH transcription factor 1; GAPDH: Glyceraldehyde 3-phosphate dehydrogenase. Results are presented as mean ± SEM, **p < 0.01.

Transwell assay was used to detect the migration and invasion of MDA-MB-415 cells. The results showed that *ANKRD22* shRNA decreased the number of migratory and invasive MDA-MB-415 cells as compared to the control group (Figure 3A). As expected, *CDH1* and *KRT18* were upregulated, whereas *CDH2*, *VIM*, and *TWIST1* were downregulated after *ANKRD22* was knocked down via qRT-PCR (Figure 3B). KRT18 protein level was elevated and VIM and TWIST1 levels were decreased as measured by immunoblot assay (Figure 3C). Subsequently, the expression levels of two EMT-related proteins, N-cadherin and E-cadherin, in MDA-MB-415 cells were analyzed (Figure 3C). We found a significant reduction of N-cadherin expression and induction of E-cadherin expression in MDA-MB-415 cells after *ANKRD22* was knocked down. These results suggested that *ANKRD22* knockdown suppresses the proliferation, invasion, and EMT of breast cancer cells.

### *ANKRD22* knockdown inhibits NuSAP1 expression and regulates Wnt/β-catenin pathway activation in breast cancer cells

**FIGURE 4:**
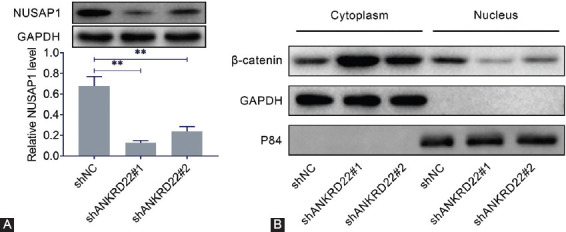
*ANKRD22* knockdown inhibits NuSAP1 expression and regulates Wnt/β-catenin pathway activation. (A) NuSAP1 protein expression levels in MDA-MB-415 cells with *ANKRD22* knockdown. (B) β-catenin protein expression level in the cytoplasm and nucleus of MDA-MB-415 cells after *ANKRD22* was knocked down. GAPDH and P84 were used as reference proteins in the cytoplasm and nucleus, respectively. *ANKRD22*: Ankyrin repeat domain 22; NuSAP1: Nucleolar and spindle-associated protein 1; GAPDH: Glyceraldehyde 3-phosphate dehydrogenase. Results are presented as mean ± SEM, **p < 0.01.

To investigate the potential mechanism underlying the effects of *ANKRD22* knockdown on breast cancer cell proliferation, invasion, and EMT, the expression level of NuSAP1, a microtubule-binding protein that participates in the cell cycle, was analyzed in MDA-MB-415 cells. *ANKRD22* knockdown significantly inhibited the expression of NuSAP1 (Figure 4A). Previously, it was demonstrated that NuSAP1 accelerates the metastasis of cervical carcinoma cells by activating Wnt/β-catenin signaling [21]. Therefore, we investigated whether ANKRD22 could regulate NuSAP1 expression by activating the Wnt/β-catenin signaling pathway in breast cancer. The results showed that *ANKRD22* knockdown remarkably increased β-catenin expression in the cytoplasm and remarkably decreased β-catenin expression in the nucleus of MDA-MB-415 cells (Figure 4B). These results indicated that *ANKRD22* knockdown positively regulated NuSAP1 expression via the Wnt/β-catenin signaling pathway.

### NUSAP1 overexpression reverses the effects of *ANKRD22* knockdown on breast cancer cell proliferation, invasion, and EMT

**FIGURE 5:**
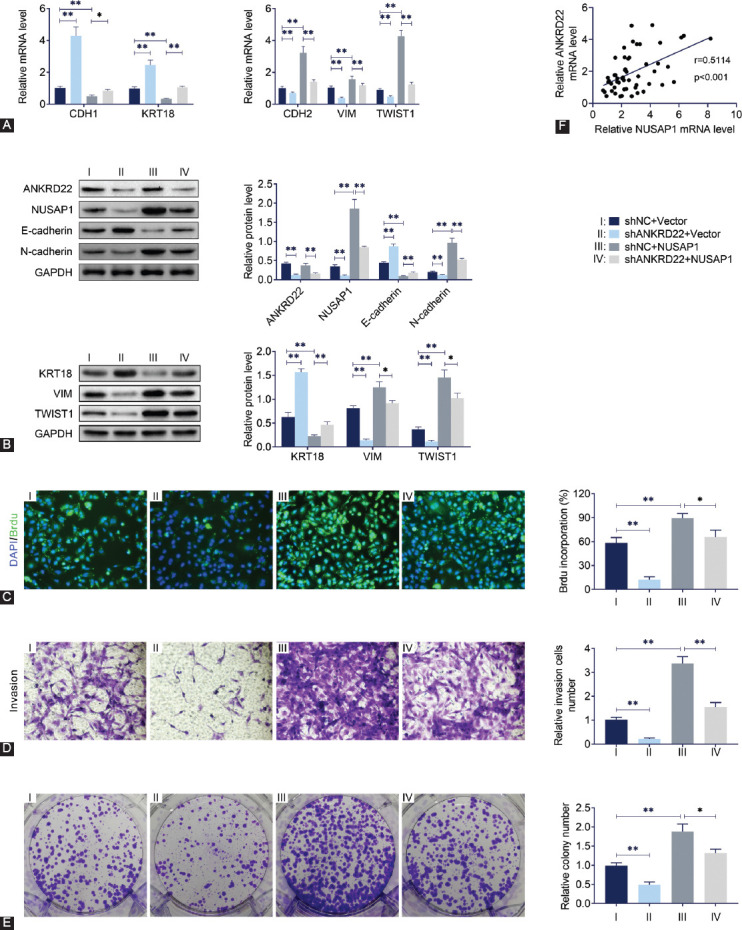
NUSAP1 overexpression reverses the effects of *ANKRD22* knockdown on breast cancer cell proliferation, invasion, and EMT. (A) The mRNA levels of *CDH1, KRT18, CDH2, VIM, and TWIST1* were detected by quantitative reverse transcription polymerase chain reaction. (B) The protein levels of *ANKRD22*, *NuSAP1*, E-cadherin, and N-cadherin were detected by immunoblotting. (C) The effects of *NUSAP1* overexpression on cell proliferation were detected by BrdU assay. (D) The effects of *NUSAP1* overexpression on cell invasion and migration were detected by transwell assay. (E) Colony formation assay was performed to detect the effect of *NUSAP1* overexpression on cell colony formation. (F) The correlation analysis between *NUSAP1* and *ANKRD22* mRNA levels in 53 patients with breast cancer. *ANKRD22*: Ankyrin repeat domain 22; NuSAP1: Nucleolar and spindle-associated protein 1; EMT: Epithelial-mesenchymal transition; CDH1: E-cadherin; CDH2: N-cadherin; KRT18: Keratin 18; VIM: Vimentin; TWIST1: Twist family bHLH transcription factor 1; GAPDH: Glyceraldehyde 3-phosphate dehydrogenase. Results are presented as mean ± SEM, *p < 0.05, **p < 0.01.

To confirm the effect of NuSAP1 on MDA-MB-415 cells, a *NUSAP1* overexpression vector was constructed. The *NUSAP1* overexpression vector and *ANKRD22* shRNA plasmids were co-transfected into breast cancer cells. qRT-PCR assays showed that overexpression of *NUSAP1* reduced the mRNA level of *CDH1* and *KRT18* and increased the mRNA levels of *CDH2*, *VIM*, and *TWIST1* (Figure 5A). Immunoblot assays confirmed the co-transfection efficiency of *NUSAP1* overexpression vector and *ANKRD22* shRNA plasmids in MDA-MB-415 cells (Figure 5B). Overexpression of *NUSAP1* reversed the expression levels of E-cadherin and N-cadherin in MDA-MB-415 cells that were caused by *ANKRD22* knockdown (Figure 5B). Moreover, overexpression of *NUSAP1* suppressed the protein level of KRT18 and facilitated the level of VIM and TWIST1 (Figure 5B). Next, we investigated whether *NUSAP1* overexpression could reverse the effects of *ANKRD22* knockdown on the proliferation, invasion, and EMT of MDA-MB-415 cells. BrdU assays confirmed that the decreased proliferative capacity of MDA-MB-415 cells caused by *ANKRD22* knockdown was significantly restored by *NUSAP1* overexpression (Figure 5C). *NUSAP1* overexpression could also reverse the effects of *ANKRD22* knockdown on invasive capacity in MDA-MB-415 cells (Figure 5D). Colony formation assays showed that *NUSAP1* overexpression reversed the effects of *ANKRD22* knockdown on the relative number of colonies in MDA-MB-415 cells (Figure 5E). qRT-PCR assay confirmed the positive correlation between the expression levels of *NUSAP1* and *ANKRD22* in breast cancer tissues from 53 patients (Figure 5F). These results indicated that overexpression of *NUSAP1* reversed the inhibitory effects of *ANKRD22* knockdown on the growth, invasion, and EMT of breast cancer cells.

## DISCUSSION

The global incidence of breast cancer remains high since the late 1970s [22]. One in eight women in the United States suffers from breast cancer during their lifetime [23]. Molecular targeted therapy is considered as one of the most promising approaches. In particular, *ANKRD22* has been previously diagnosed as a marker for pancreatic cancer, pancreatic ductal cancer, and NSCLC [24,25]. However, its biological role in breast cancer is still unclear. Further understanding of the molecular mechanisms of *ANKRD22* in the onset and progression of breast cancer is particularly important for developing new strategies for treating patients with metastatic and recurrent breast cancer.

The present study initially explored the possible involvement of *ANKRD22* in the progression and development of breast cancer through analyzing *ANKRD22* mRNA expression levels in 70 clinical samples. The level of *ANKRD22* expression in breast cancer tissue was higher than that in normal breast tissue. Moreover, breast cancer patients with higher *ANKRD22* expression had lower survival rates compared to those with lower *ANKRD22* expression. These results suggest that *ANKRD22* may regulate the proliferation and metastasis of breast cancer cells. The expression of *ANKRD22* in four different breast cancer cell lines was also measured. We found that MDA-MB-415 cell line had the highest *ANKRD22* expression and the proliferation, migration, and invasion of MDA-MB-415 cells were significantly inhibited by *ANKRD22* knockdown, consistent with the reduced expression of N-cadherin and increased expression of E-cadherin. E-cadherin is a calcium-dependent transmembrane cell adhesion molecule, which plays a key role in maintaining epithelial polarity and integrity. Previous studies proved that loss of E-cadherin could promote the invasion and metastasis of tumor, which was also related to poor prognosis in patients with breast, colon, and other cancers [26,27]. The results from the current study showed that ANKRD22 contributed to breast cancer progression, indicating that it might act as a potential marker for the therapy of breast cancer.

NuSAP1 is a microtubule-binding protein involved in several types of cancers such as prostate cancer, colorectal cancer, and astrocytoma [28-30]. Previous studies found that high expression of NuSAP1 was significantly correlated with poor prognosis of breast cancer and melanoma [17]. NuSAP1 was also previously reported to be highly expressed in breast cancer and involved in breast cancer progression [18]. In this study, *ANKRD22* knockdown inhibited the expression of NuSAP1 and increased β-catenin expression in MDA-MB-415 cells. Moreover, overexpression of *NUSAP1* reversed the inhibitory effects of *ANKRD22* knockdown on the proliferation, invasion, and EMT of breast cancer cells. A previous study indicated that ANKRD22 could serve as a transcription factor and activate the expression of E2F1 in lung cancer progression [24], which suggests that ANKRD22 might regulate other factors to affect breast cancer development and this needs further investigation.

The Wnt/β-catenin pathway plays a key role in the progression of various types of cancers, which contributes to the metastasis of cancer [31]. NuSAP1 has been demonstrated to activate Wnt/β-catenin signaling in cervical cancer cells [21]. In this study, *CDH1* and *KRT18* were upregulated, whereas *CDH2*, *VIM*, and *TWIST1* were downregulated after *ANKRD22* was knocked down. We found a significant reduction of N-cadherin expression and induction of E-cadherin expression in MDA-MB-415 cells after *ANKRD22* was knocked down. Besides, *NUSAP1* overexpression could reverse the effects of *ANKRD22* knockdown on these effects. Therefore, we concluded that high expression of ANKRD22 could facilitate the progress of breast cancer, at least partially, by activating Wnt/β-catenin signaling via NuSAP1.

## CONCLUSION

**FIGURE 6:**
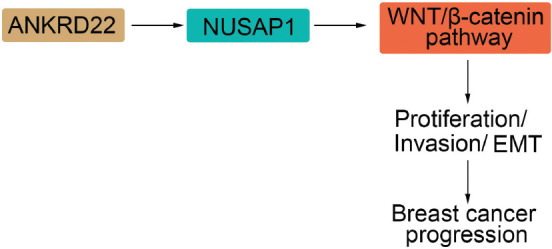
The regulatory mechanism underlying the effects of ANKRD22 on breast cancer cell growth. ANKRD22 enhanced breast cancer cell growth by activating the Wnt/β-catenin pathway via modulating NuSAP1 expression. ANKRD22: Ankyrin repeat domain 22; NuSAP1: Nucleolar and spindle-associated protein 1.

Our study demonstrated that ANKRD22 was dramatically upregulated in both breast cancer tissues and cell lines. *ANKRD22* knockdown suppressed the proliferation, invasion, and EMT of breast cancer cells and the underlying mechanism was through regulating NuSAP1. These findings might provide a clue for a better understanding of the occurrence and progression of breast cancer (Figure 6). Hence, ANKRD22 may be a valuable diagnostic marker for breast cancer.
